# DeepLMI: deep feature mining with a globally enhanced graph convolutional network for robust lncRNA–miRNA interaction prediction

**DOI:** 10.1093/bioinformatics/btag145

**Published:** 2026-03-26

**Authors:** Zhijian Huang, Kai Chen, Xianshu Wang, Junheng Wang, Siyuan Shen, Yuanpeng Zhang, Min Wu, Lei Deng

**Affiliations:** School of Computer Science and Engineering, Central South University, Changsha 410083, China; School of Computer Science and Engineering, Central South University, Changsha 410083, China; School of Computer Science and Engineering, Central South University, Changsha 410083, China; School of Computer Science and Engineering, Central South University, Changsha 410083, China; School of Computer Science and Engineering, Central South University, Changsha 410083, China; School of Computer Science and Engineering, Central South University, Changsha 410083, China; Institute for Infocomm Research, Agency for Science, Technology and Research (A*STAR), Singapore 138632, Singapore; School of Computer Science and Engineering, Central South University, Changsha 410083, China

## Abstract

**Motivation:**

Interactions between long noncoding RNAs (lncRNAs) and microRNAs (miRNAs) play pivotal roles in gene regulation and disease progression, notably through mechanisms such as competitive miRNA sponging. Accurate identification of lncRNA–miRNA interactions is therefore essential for understanding disease mechanisms and discovering therapeutic targets. However, current knowledge is largely derived from labor-intensive and costly biological experiments, underscoring the need for reliable computational approaches.

**Results:**

We propose DeepLMI, a novel deep learning framework for lncRNA–miRNA interaction prediction that integrates deep feature mining with a globally enhanced graph convolutional network. To effectively capture the distinct properties of lncRNAs and miRNAs, DeepLMI employs specialized feature extraction modules: for lncRNAs, we combine sequence pretraining with self-attention mechanisms to learn multiscale semantic representations; for miRNAs, we fuse heterogeneous features through a graph convolutional encoder. To further address the sparsity and structural complexity of known RNA interaction networks, we design a Global-Enhanced Graph Convolutional Network that jointly models local neighborhood information and global topological signals. The embeddings learned for lncRNAs and miRNAs are then integrated to infer interaction probabilities. Extensive experiments across multiple datasets and evaluation settings demonstrate that DeepLMI consistently outperforms existing state-of-the-art methods and exhibits strong robustness, highlighting its potential as a valuable tool for RNA interaction analysis and disease research.

**Availability and implementation:**

The codes and data are publicly available at https://github.com/Hhhzj-7/DeepLMI.

## 1 Introduction

Long noncoding RNAs (lncRNAs) and microRNAs (miRNAs) are two major classes of regulatory noncoding RNAs that orchestrate gene expression in cells. Notably, over 98% of transcribed RNA in humans does not encode proteins, and lncRNAs (transcripts >200 nucleotides) as well as miRNAs (∼22-nt small RNAs) form key parts of this pervasive noncoding transcriptome (Wang *et al.* 2022). As important regulators, lncRNAs control gene expression at multiple levels, ranging from epigenetic chromatin modification to regulation at both the transcriptional and posttranscriptional levels (Wang *et al.* 2023a). In contrast, miRNAs typically guide Argonaute-silencing complexes to complementary sites on target mRNAs, causing mRNA degradation or translational repression. Indeed, it is estimated that the 2600 known human miRNAs collectively regulate more than 60% protein-coding genes, thus influencing virtually all biological pathway (Shu *et al.* 2017). A pivotal regulatory mechanism underlying the crosstalk between lncRNAs and miRNAs is the competing endogenous RNA (ceRNA) network, whereby lncRNAs bearing miRNA response elements can sequester miRNAs and thereby attenuate their suppressive effects on common target mRNAs (Wang *et al.* 2023a). This lncRNA–miRNA crosstalk is now recognized as a fundamental aspect of gene regulatory networks and has been implicated in diverse diseases. For instance, the maternally imprinted lncRNA H19 acts as a molecular sponge for the tumor-suppressor let-7 miRNA, thereby derepressing let-7 target oncogenes and promoting cancer metastasis (Peng *et al.* 2017). Similarly, lncRNA MIAT sequesters miR-149-5p, upregulating the pro-atherogenic receptor CD47 and exacerbating cardiovascular pathogenesis in atherosclerosis (Ye *et al.* 2019). In neurodegenerative disease, the brain-expressed BACE1-AS lncRNA was shown to elevate the Alzheimer’s disease-related BACE1 gene by absorbing multiple BACE1-targeting miRNAs and preventing their silencing effect (Zeng *et al.* 2019). The ceRNA mechanism is also implicated in immune and inflammatory disorders. For example, lncRNA NEAT1 enhances pathogenic Th17 cell responses in autoimmune diseases by sequestering miR-128-3p (Chen *et al.* 2023). As lncRNA–miRNA crosstalk plays a central role in gene regulation and disease, accurate prediction of lncRNA–miRNA interactions is essential for uncovering their functional implications. However, identifying lncRNA–miRNA interactions solely through laboratory experiments is extremely challenging and labor-intensive, and only a small fraction of the potential lncRNA–miRNA interactions have been validated to date (Wang *et al.* 2022). These limitations underscore the need for computational approaches, which are being applied to a growing number of RNA-related challenges (Huang *et al.* 2025), to systematically predict lncRNA–miRNA interactions and accelerate the discovery of novel regulatory interactions.

Current computational methods for predicting lncRNA–miRNA interactions primarily include traditional machine learning approaches and deep learning models. Early machine learning methods typically relied on predefined similarity metrics, network propagation, or matrix factorization to infer potential interactions, such as EPLMI (Huang *et al.* 2018b), SLNPM (Zhang *et al.* 2019), and LMFNRLMI (Liu *et al.* 2020). Although these approaches provided valuable insights, their dependence on manually designed features and static similarity measures often limited their generalization ability. Recently, deep learning methods have enabled automatic representation learning from RNA sequences and interaction graphs. For example, preMLI (Yu *et al.* 2022) leveraged unsupervised rna2vec embeddings, SPGNN (Wang *et al.* 2023b) refined *k*-mer-based Doc2Vec representations using GNNs, and MPGK-LMI (Xie *et al.* 2024) integrated meta-path similarity with graph attention mechanisms.

Although these advances have been made, several limitations still constrain current methods. (i) Due to the diverse and complex sequence patterns of lncRNAs, many methods rely on *k*-mer embeddings, yet the use of a single *k* often fails to capture multiscale semantic information. (ii) Feature extraction strategies for miRNAs are frequently analogous to those used for lncRNAs, overlooking their unique biological properties and multisource feature space. (iii) The sparsity of known lncRNA–miRNA interactions impedes conventional GCNs from effectively aggregating long-range topological information. (iv) Existing studies lack rigorous evaluation under cold-start or independent dataset scenarios, limiting insights into model generalizability and robustness.

To overcome these limitations, we propose DeepLMI, a deep learning framework that integrates tailored feature mining with a Global-Enhanced Graph Convolutional Network (GE-GCN) for accurate lncRNA–miRNA interaction prediction. For lncRNAs, we design a multiscale sequence encoder that combines pretrained representations with self-attention to capture fine-grained semantic patterns. For miRNAs, we integrate secondary-structure graphs and embeddings learned from an RNA language model to incorporate diverse biological cues. We then build an interaction graph based on known interactions and introduce a GE-GCN that complements neighborhood aggregation with global information exchange, enabling the model to capture both local dependencies and higher order signals. Finally, the learned embeddings are fused and fed into a fully connected layer to infer interaction probabilities. Extensive cross-validation, cold-start, and independent-test experiments demonstrate that DeepLMI consistently outperforms state-of-the-art methods, exhibiting strong robustness and the ability to predict novel lncRNA–miRNA interactions.

## 2 Materials and methods

### 2.1 Overview

**Figure 1:**
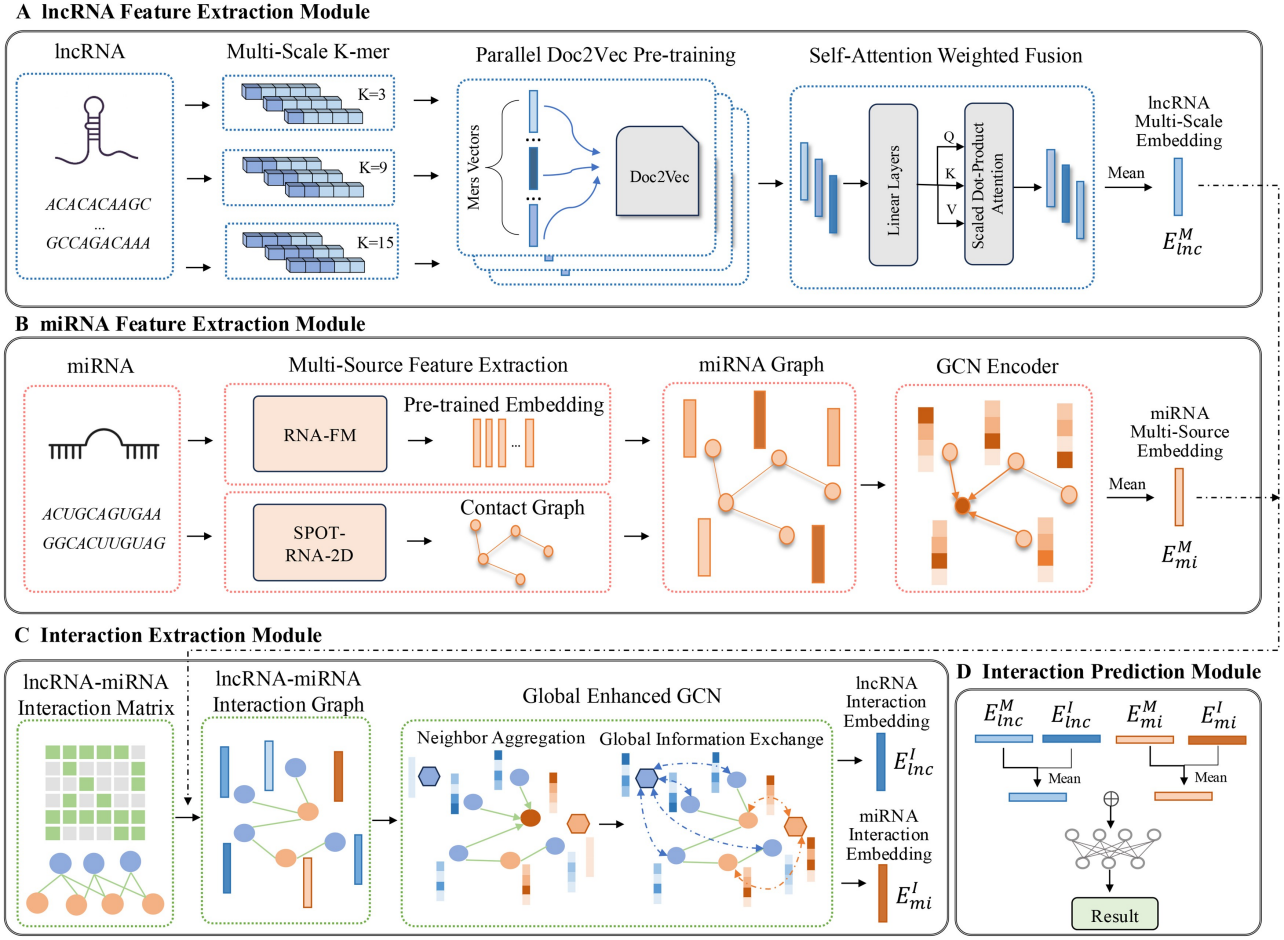
Overview of the DeepLMI framework. (A, B) Feature extraction modules for lncRNA and miRNA. (C) lncRNA–miRNA interaction extraction module to integrate RNA and small molecule information from different views. (D) lncRNA–miRNA interaction prediction module to combine the lncRNA and miRNA embeddings from (A), (B), and (C).

As illustrated in Fig. 1, DeepLMI comprises four modules. For lncRNA, we designed a multiscale sequence semantic information extraction module. The *k*-mer method can divide the lncRNA sequence into overlapping subsequences of length *k*. We performed parallel pretraining processes with multiple *k*-values using Doc2Vec (Le and Mikolov 2014), generating multiscale lncRNA semantic embeddings. Then, we employed a self-attention mechanism to adaptively fuse multiscale semantic information. For miRNA, we designed a multisource feature extraction module. We first obtained the predicted secondary structure of miRNA using the SPOT-RNA-2D (Singh *et al.* 2022), and extracted pretrained embeddings from RNA-FM (Chen *et al.* 2022). Then, we constructed a contact graph based on the predicted secondary structure, where the edges represent structural contacts and the nodes are initialized with the pretrained embeddings. A GCN (Kipf and Welling 2016) was subsequently applied to encode the multisource features of miRNA. To explore the complex relationship between lncRNAs and miRNAs, we proposed a GE-GCN. Based on known lncRNA–miRNA interactions, we constructed an interaction graph. To enable efficient extraction of interaction information under sparse conditions, we introduced a central node for both lncRNA and miRNA, respectively, to facilitate global information exchange between the two types of RNA. Finally, the lncRNA–miRNA interaction prediction module integrated the embeddings of lncRNA and miRNA with their interaction graph representations. These embeddings were fed into a fully connected layer to perform lncRNA–miRNA interaction prediction.

### 2.2 Datasets and experimental settings

To ensure a robust evaluation, we assessed our model using two distinct datasets under three experimental settings. The cross-validation is constructed based on the dataset from Wang *et al.* (2023b), after removing redundant interaction entries to ensure higher data consistency and reliability. It comprises 284 unique lncRNAs, 517 miRNAs, and 1057 experimentally verified lncRNA–miRNA interactions. The external dataset was independently constructed to assess the model’s generalization capability. It includes 472 lncRNAs, 295 miRNAs, and 890 interaction pairs from RNAcentral (RNAcentral Consortium 2021).

To comprehensively assess the predictive performance, we designed three groups of experimental settings: standard cross-validation, blind test on cross-validation dataset, and independent test. For the standard cross-validation setting, we conducted 5-fold cross-validation on the cross-validation dataset, ensuring that each fold maintains different lncRNA–miRNA interaction values. For the blind test setting, we constructed three distinct dataset partitions based on 5-fold cross-validation: blind lncRNAs, blind miRNAs, and blind both lncRNAs and miRNAs. For each of these partitions, 5-fold cross-validation was performed based on the designated entity. In each fold, interactions involving entities of the blind type assigned to the validation set were used for validation, while all remaining interactions were used for training. Detailed definitions and data conditions are provided in the Supplementary Material, available as supplementary data at *Bioinformatics* online. For independent test setting, the model was trained on the cross-validation dataset and subsequently validated and tested on the external dataset.

We compared DeepLMI against several state-of-the-art baselines, including preMLI, SPGNN, HGCLMDA (Hu *et al.* 2023), MNCLCDA (Li *et al.* 2023), and MPGK-LMI. Detailed descriptions of the baseline methods are available in the Supplementary Material, available as supplementary data at *Bioinformatics* online. The evaluation metrics include area under the ROC curve (AUC), area under the precision–recall curve (AUPR), F1-score, precision (Pre), recall (Rec), and normalized discounted cumulative gain (NDCG). All experiments were conducted on the same server with a single NVIDIA RTX A6000 GPU. We employed the Adam optimizer with a learning rate of 1e-4. The model was trained for up to 200 epochs on each cross-validation fold, using the binary cross-entropy with logits loss as the loss function.

### 2.3 Feature extraction module for lncRNA

#### 2.3.1 Parallel Doc2Vec pretraining

The lncRNA sequences are typically long, and their biological functions are often determined by multiple local structural features and broader contextual sequence information. Consequently, single-scale feature extraction methods are insufficient to comprehensively capture their underlying biological patterns. To address this challenge, we proposed a parallel Doc2Vec pretraining strategy that integrates multiscale *k*-mer segmentation with Doc2Vec to comprehensively explore the latent biological information within lncRNA sequences.

First, we segmented each lncRNA sequence using sliding windows of multiple sizes to generate multiscale *k*-mer representations (e.g. *k* = 3, 9, 15). The *k*-mer approach enables us to decompose the lncRNA into fixed-length subsequences, which effectively capture local sequence patterns. Specifically, for an lncRNA sequence *S*_lnc_ of length *L*_lnc_, a sliding window of size *k* produces $\left(L_{\mathrm{lnc}}-k+1\right)$ consecutive *k*-mer fragments.

By incorporating *k*-mer representations with multiple *k*-values, we are able to capture local features of lncRNA sequences at different granularities. This multiscale strategy enhances the model’s ability to recognize informative segments of varying lengths within lncRNAs. Consequently, we obtained a collection of lncRNA segments of varying lengths, analogous to the Bag-of-words in natural language processing. To capture the contextual relationships and sequential patterns among *k*-mers, we employed the Doc2Vec model to perform unsupervised training on all lncRNA segments. Doc2Vec is an embedding algorithm designed to learn document representations, capable of mapping variable-length word sequences into fixed-length vectors. Each lncRNA sequence is treated as a document, while the lncRNA segments are regarded as words within that document. Different *k*-values in *k*-mer produce lncRNA documents at varying segment scales. For the vocabulary of lncRNA generated by multiple *k*-values, we applied the Distributed Memory Model of Paragraph Vectors (PV-DM) in parallel for pretraining, thereby obtaining lncRNA embeddings at different scales. PV-DM aims to jointly learn representations of individual *k*-mers and the entire sequence by predicting context. Given a context window of size *c*, the hidden representation $h_{t}^{k}$ for position *t* is computed as the average of the surrounding *k*-mer embeddings and the document embedding:


(1) $$h_{t}^{k}=\frac{1}{2c+1}\left(E_{\text{lnc}}^{k}+\sum_{\begin{array}{c} -c\leq j\leq c\\ j\neq 0 \end{array}}w_{t+j}^{k}\right),$$

where $w_{t+j}^{k}$ is the embedding of the *k*-mer of size *k* at position t+j, and $E_{\mathrm{lnc}}^{k}$ is the learned embedding vector representing the entire lncRNA sequence. The model then predicts the probability of the target *k*-mer $w_{t}^{k}$ using a softmax classifier:


(2) $$P(w_{t}^{k}|D_{\mathrm{lnc}})=\frac{\;\text{exp}(v_{w_{t}^{k}}^{⊤}h_{t}^{k})}{\sum_{w^{k}\in V^{k}}\;\text{exp}(v_{w^{k}}^{⊤}h_{t}^{k})},$$

where $D_{\mathrm{lnc}}$ denotes the combined condition, consisting of the lncRNA document vector and the local context window of *k*-mers around the target $w_{t}^{k}$, $v_{w^{k}}$ represents the context-prediction vector for $w^{k}$ used in the output layer of the PV-DM model and $V^{k}$ is the lncRNA vocabulary containing all unique *k*-mers of size *k*. The entire model was trained by maximizing the log-likelihood over all center positions across all sequences, and negative sampling is used to optimize training efficiency. After training, document embeddings $E_{\text{lnc}}^{k}$ served as representations of the input lncRNA sequences, capturing both local nucleotide dependencies and contextual semantics at the higher level. As we processed the lncRNA sequences using *k*-mers of different sizes, we can perform parallel pretraining to obtain lncRNA embeddings with different levels of information granularity.

#### 2.3.2 Self-attention weighted fusion

To adaptively integrate lncRNA representations of different levels of information level, we introduced a self-attention-based feature fusion mechanism. Self-attention adaptively generates contextualized representations by weighting and aggregating features across the entire sequence, where the weights are computed from the compatibility of query and key vectors (Vaswani *et al.* 2017). Here, we employed a self-attention mechanism to adaptively fuse lncRNA embeddings with information at different scales. The stacked LncRNA embeddings from different scales are denoted as $H_{\text{lnc}}$. Query *Q*_lnc_, key *K*_lnc_, and value *V*_lnc_ matrices are obtained via linear projections. The multiscale fusion is achieved by scaled dot-product attention to obtain the lncRNA representations $E_{\text{lnc}}^{M}$:


(3) $$E_{\text{lnc}}^{M}=\text{Mean}\left(\text{Softmax}\left(\frac{Q_{\text{lnc}}K_{\text{lnc}}{\;}^{⊤}}{\sqrt{d}}\right)V_{\text{lnc}}\right),$$

where Softmax(·) is applied row-wise to normalize the attention scores, *d* is the hidden size and Mean(·) denotes mean pooling operation.

### 2.4 Feature extraction module for miRNA

#### 2.4.1 Multisource feature extraction

Unlike lncRNA, which have longer sequences and diverse structures, miRNAs are a class of short noncoding RNAs whose functions are highly dependent on conserved sequence patterns and stable secondary structures. Therefore, to accurately capture the biological properties that determine their function, we designed a specialized multisource feature extraction module for miRNA, aiming to deeply integrate the semantic information of their sequences with the predicted topological structure.

Conventional sequence embeddings fail to adequately represent nucleotide-level context (Huang *et al.* 2024). We therefore adopted RNA-FM (Chen *et al.* 2022), a transformer-based language model pretrained on millions of noncoding RNA sequences. Through unsupervised pretraining, RNA-FM captures distributed representations that encode local sequence motifs and their contextual dependencies. For an miRNA sequence of length $L_{\text{mi}}$, we imputed it into the pretrained RNA-FM to generate a high-dimensional embedding vector for each nucleotide in the sequence. These vectors encoded the identity of the nucleotide and reflect its functional semantics in the sequence context, forming the initial node embedding $h_{\text{mi}}^{0}$ of the subsequent graph model.

The function of an miRNA is closely tied to the structural patterns captured in its contact map. To provide the model with more detailed information, we employed SPOT-RNA-2D (Singh *et al.* 2022), a deep learning-based predictor, to generate secondary-structure contact maps that capture both canonical Watson-Crick pairs and noncanonical interactions. The predicted contacts are encoded as a symmetric binary adjacency matrix $A_{\text{mi}}\in {\left\{0,1\right\}}^{L_{\text{mi}}\times L_{\text{mi}}}$, where $A_{\text{mi}}(i,j)=1$ denotes spatial proximity between nucleotides *i* and *j*. This adjacency matrix provides the essential topological framework for subsequent graph convolution operations.

#### 2.4.2 Graph convolutional network

After obtaining the initial node embedding and graph topology, we used a GCN to learn a comprehensive miRNA feature representation that integrates multisource information. The core idea of GCN is to update the node representation by iteratively aggregating feature information from each node’s neighborhood (Kipf and Welling 2016). For a GCN model, the propagation rule for its *p*th layer can be formally defined as


(4) $$H_{\text{mi}}^{p+1}=\sigma \left({\tilde{D}}_{\text{mi}}^{-\frac{1}{2}}{\hat{A}}_{\text{mi}}{\tilde{D}}_{\text{mi}}^{-\frac{1}{2}}h_{\text{mi}}^{p}W^{p}\right),$$

where ${\hat{A}}_{\text{mi}}=A_{\text{mi}}+I$ is an adjacency matrix with self-loops added to ensure that nodes consider their own information when aggregating neighbor information, ${\tilde{D}}_{\text{mi}}$ is the diagonal matrix of ${\hat{A}}_{\text{mi}}$, $h_{\text{mi}}^{p}$ is the node feature matrix for the *p*th layer of the GCN, $W^{p}$ is the trainable weight matrix for this layer, and σ is the nonlinear activation function. After *p* convolutional layers, we performed mean pooling on the final embedding vectors of all nucleotide nodes to obtain the embedding of the entire miRNA molecule. The resulting vector $E_{\text{mi}}^{M}$ is the miRNA multisource embedding, which can comprehensively encode miRNA and serve as its biological feature embedding input into the subsequent interaction prediction module.

### 2.5 Interaction extraction module

#### 2.5.1 Graph construction for lncRNA–miRNA interactions

To learn expressive feature representations from known lncRNA–miRNA interactions, we designed an interaction extraction module to capture their underlying topological patterns. The first step is to construct a graph that formally describes the connections between lncRNAs and miRNAs. First, we constructed a heterogeneous bipartite graph to model lncRNA–miRNA interactions, denoted as $G^{I}=(U,C)$. The graph comprises two disjoint node sets: a set of *N* lncRNAs, $U_{\text{lnc}}$, and a set of *M* miRNAs, $U_{\text{mi}}$. The complete node set is defined as their union, $U=U_{\text{lnc}}\cup U_{\text{mi}}$. The edge set *C*, representing the known interactions, is encoded by an adjacency matrix $A^{I}\in {\left\{0,1\right\}}^{N\times M}$. An edge (u,v) exists between an lncRNA $u\in U_{\text{lnc}}$ and an miRNA $v\in U_{\text{mi}}$ if and only if an interaction between them has been experimentally verified, i.e. $A_{uv}^{I}=1$. With few known interactions, the constructed graph is inherently sparse.

#### 2.5.2 Global-enhanced graph convolutional network

The scarcity of experimentally verified data on lncRNA–miRNA interactions results in highly sparse interaction maps. This sparsity severely impairs effective information propagation through neighbor aggregation in standard GCNs. To address this limitation, we proposed a GE-GCN. This network augments the traditional local information propagation mechanism with a global information exchange layer, addressing the problem that conventional GCN models are difficult to train in sparse graph environments.

The essence of GE-GCN is to update node representations through a two-stage information propagation process: local propagation and global exchange. The local propagation stage follows the conventional paradigm of GCN, whose propagation rule has been defined in detail in Equation (4). This operation aggregates information from the immediate neighbors of each node. However, its effectiveness is significantly reduced in sparse graphs where most nodes have only a few connections. Standard message passing fails in sparse regimes where receptive fields remain limited even after multiple hops. Our global exchange mechanism addresses this by computing type-specific summary statistics that are broadcast to all nodes within each molecular class. This stage involves two virtual global nodes: a global lncRNA node $g_{\text{lnc}}$ and a global miRNA node $g_{\text{mi}}$. These nodes act as information hubs for their respective lncRNA and miRNA populations. Specifically, in each layer of GE-GCN, after the local propagation step is completed, a global information exchange operation is conducted. This process begins with a gathering step to form a global contextual representation. As shown in Equation (5), the global lncRNA node aggregates feature vectors from all lncRNA nodes, while the global miRNA node does similar:


(5) $$h_{g\_\text{lnc}}^{l+1}=\frac{1}{N}\sum_{u\in U_{\text{lnc}}}h_{u}^{l+1},h_{g\_\text{mi}}^{l+1}=\frac{1}{M}\sum_{v\in U_{\text{mi}}}h_{v}^{l+1},$$

Here, $h_{u}^{l+1}$ and $h_{v}^{l+1}$ denote the node feature vectors after the local propagation phase. Subsequently, in the broadcast and update step, these global contextual representations are broadcast back to all corresponding nodes. Finally, we can generate the interaction topology embeddings ($E_{\text{lnc}}^{I}$ and $E_{\text{mi}}^{I}$) for lncRNAs and miRNAs.

### 2.6 Interaction prediction module

After obtaining both the biological feature embeddings and the interaction topology embeddings for lncRNAs and miRNAs, we designed an interaction prediction module to integrate this multiperspective information and to score the potential interaction between lncRNA–miRNA pairs. We first fused their fine-grained embeddings($E_{\text{lnc}}^{M}$ and $E_{\text{mi}}^{M}$) and interaction topology embeddings($E_{\text{lnc}}^{I}$ and $E_{\text{mi}}^{I}$) by mean operation, separately. Then, we concatenated these two embeddings and fed them into a multilayer perceptron to obtain the predicted interaction score. More details can be found in the Supplementary Material, available as supplementary data at *Bioinformatics* online.

## 3 Results

### 3.1 Performance under standard setting

As shown in Table 1, DeepLMI achieved the best overall performance across all evaluation metrics under the standard cross-validation setting, with the F1-score of 0.901, the AUC of 0.952, AUPR of 0.945, and the NDCG of 0.991. Compared with the second best-performing method, MPGK-LMI, DeepLMI improved AUC by 8.4% and AUPR by 12.4%, demonstrating its superior ability to capture both sequence and topological features. Notably, our method also reached the highest recall (0.967), which indicates excellent sensitivity in identifying true interactions, while maintaining strong precision (0.843). These results highlight that DeepLMI is capable of not only accurately identifying but also predicting the vast majority of lncRNA–miRNA interactions. More results can be found in the Supplementary material, available as supplementary data at *Bioinformatics* online.

**Table 1:** Performance comparison under standard setting.

Methods	F1	AUC	AUPR	NDCG	Pre	Rec
preMLI	0.640	0.646	0.681	0.913	0.596	0.753
SPGNN	0.760	0.844	0.849	0.972	0.800	0.730
HGCLMDA	0.591	0.648	0.683	0.931	0.618	0.566
MNCLCDA	0.672	0.668	0.723	0.945	0.531	0.920
MPGK-LMI	0.888	0.878	0.841	0.965	**0.875**	0.902
DeepLMI	**0.901**	**0.952**	**0.945**	**0.991**	0.843	**0.967**

Note: The best performance for each metric is marked in bold, while the second-best performance is marked in underlined.

### 3.2 Performance under blind test setting

To assess the generalization capability under realistic scenarios, we designed three blind test settings: blind lncRNA, blind miRNA, and blind both lncRNA and miRNA. The results of blind lncRNA are shown in Table 2, DeepLMI obtained the highest F1-score (0.903) and AUC (0.937), outperforming MPGK-LMI and MNCLCDA by significant margins. While MNCLCDA performs marginally better in AUPR and precision, its significantly lower recall indicates limited sensitivity. In contrast, our method maintains high precision while substantially improving recall, achieving a more robust and comprehensive performance across all evaluation metrics.

**Table 2:** Performance comparison under blind lncRNA setting.

Methods	F1	AUC	AUPR	NDCG	Pre	Rec
preMLI	0.565	0.543	0.550	0.574	0.547	0.675
SPGNN	0.167	0.356	0.447	0.836	0.664	0.149
HGCLMDA	0.526	0.580	0.566	0.869	0.575	0.488
MNCLCDA	0.820	0.878	**0.904**	**0.984**	**0.866**	0.781
MPGK-LMI	0.682	0.719	0.595	0.902	0.676	0.708
DeepLMI	**0.903**	**0.937**	0.891	0.955	0.854	**0.961**

Note: The best performance for each metric is marked in bold, while the second-best performance is marked in underlined.

In the blind miRNA scenario (Table 3), DeepLMI outperformed all baselines, reaching the F1 of 0.913 and the AUC of 0.963. Compared with MNCLCDA, which achieved the second-best performance (F1 = 0.824, AUC = 0.870), our model demonstrated relative improvements of 10.8% and 10.7%, respectively. This result validates that DeepLMI captures transferable miRNA representations that enable robust prediction on unseen miRNAs.

**Table 3:** Performance comparison under blind miRNA setting.

Methods	F1	AUC	AUPR	NDCG	Pre	Rec
preMLI	0.624	0.601	0.648	0.847	0.600	0.691
SPGNN	0.279	0.420	0.508	0.860	0.659	0.188
HGCLMDA	0.583	0.654	0.671	0.921	0.634	0.540
MNCLCDA	0.824	0.870	0.903	0.984	**0.897**	0.768
MPGK-LMI	0.711	0.741	0.645	0.919	0.738	0.698
DeepLMI	**0.913**	**0.963**	**0.957**	**0.993**	0.853	**0.983**

Note: The best performance for each metric is marked in bold, while the second-best performance is marked in underlined.

The blind setting for both lncRNA and miRNA removes overlap on both sides, meaning neither lncRNA nor miRNA in the test set appears in training. As shown in Table 4, DeepLMI achieved the highest F1 (0.944) and AUC (0.944), surpassing MPGK-LMI by 21.5% and 34.7%, respectively. These results strongly demonstrate the robustness of our method in modeling unseen entities and highlight its potential for real-world application where novel RNAs are encountered.

**Table 4:** Performance comparison under blind both lncRNA and miRNA setting.

Methods	F1	AUC	AUPR	NDCG	Pre	Rec
preMLI	0.563	0.575	0.587	0.602	0.573	0.648
SPGNN	0.533	0.556	0.588	0.846	0.681	0.540
HGCLMDA	0.601	0.659	0.694	0.907	0.667	0.556
MNCLCDA	0.687	0.598	0.211	0.376	0.048	0.212
MPGK-LMI	0.777	0.701	0.782	0.951	0.826	0.737
DeepLMI	**0.944**	**0.944**	**0.918**	**0.970**	**0.905**	**0.991**

Note: The best performance for each metric is marked in bold, while the second-best performance is marked in underlined.

Overall, DeepLMI consistently achieves leading performance across all blind test scenarios by jointly modeling lncRNA-specific, miRNA-specific, and interaction-level information. In blind lncRNA settings, the lncRNA feature extraction module that learns multiscale embeddings plays a central role by capturing hierarchical and contextual characteristics of unseen lncRNAs. In blind miRNA settings, the miRNA feature extraction module generate fine-grained multisource embeddings to represent unseen miRNAs. When both lncRNAs and miRNAs are unseen, the two feature extraction modules act synergistically to enable effective generalization. Across all blind test scenarios, the interaction extraction module contributes by modeling the overall lncRNA–miRNA interaction context, further enhancing robustness and leading to significant improvements over baseline methods.

### 3.3 Performance under independent setting

**Figure 2:**
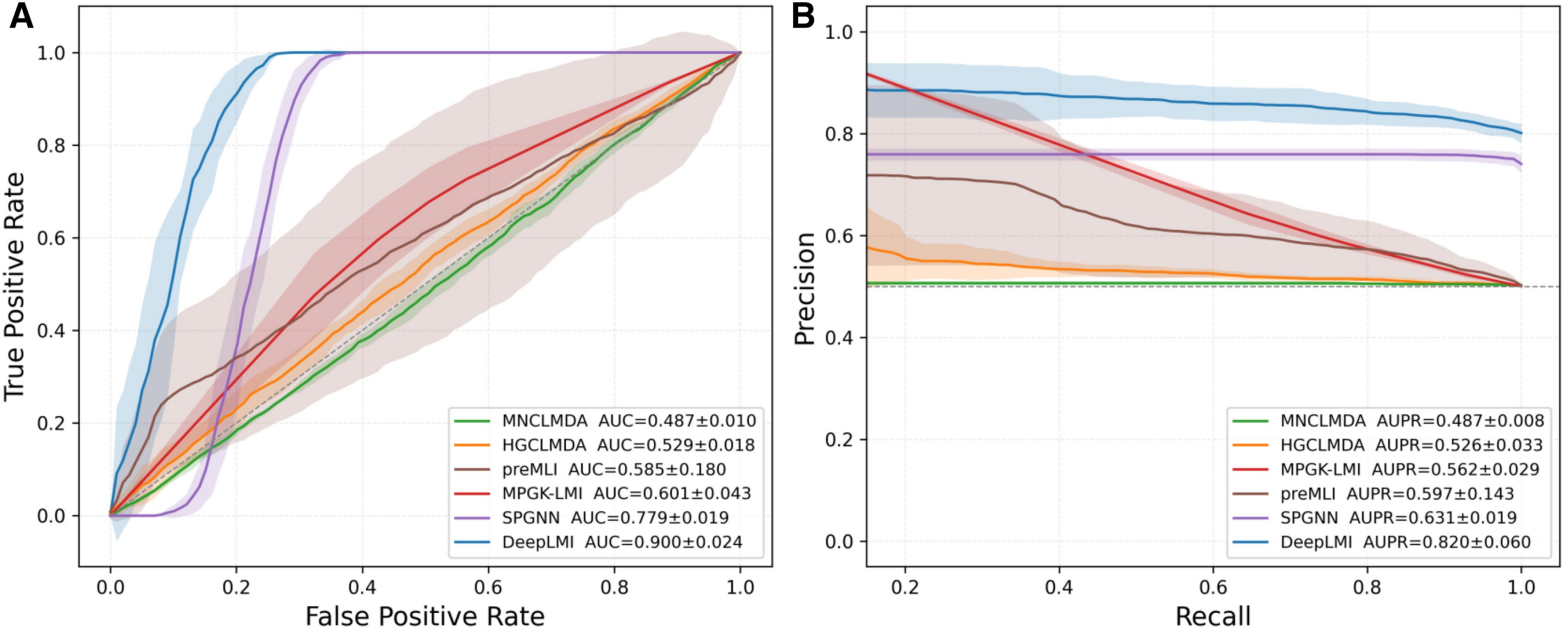
Comparison of ROC and PR curves between our model and baseline methods under independent setting. (A) ROC curve. (B) PR curve.

To further evaluate robustness across data sources, we conducted an independent test (Table 5). DeepLMI consistently outperformed competing methods, achieving the F1 of 0.889, the AUC of 0.900, and the AUPR of 0.820. Compared to the best-performing baseline SPGNN (F1 = 0.855, AUC = 0.779, AUPR = 0.631), our model improved F1, AUC and AUPR by 4.0%, 15.5% and 30.0%, demonstrating superior predictive capability in both overall ranking performance and precision–recall balance. Moreover, DeepLMI shows strong generalization capability, achieving optimal performance in cross-validation and maintaining robust results on external datasets. This consistency underscores its reliability and practical potential for real-world applications. Figure 2 presents the comparison of ROC and PR curves between our model and the baseline methods on the independent test set. The shaded areas reflect the performance variation. Our model outperforms all baselines in both ROC and PR analyses, showing higher average AUC and AUPR values. The narrow shaded regions around our curves indicate low variance, demonstrating that our model not only achieves superior predictive accuracy but also exhibits excellent stability across repeated experiments.

**Table 5:** Performance comparison on independent test set.

Methods	F1	AUC	AUPR	NDCG	Pre	Rec
preMLI	0.663	0.585	0.597	0.904	0.599	0.789
SPGNN	0.855	0.779	0.631	0.880	0.750	**0.994**
HGCLMDA	0.552	0.529	0.526	0.890	0.539	0.608
MNCLCDA	0.648	0.487	0.487	0.878	0.506	0.906
MPGK-LMI	0.650	0.601	0.562	0.875	0.589	0.744
DeepLMI	**0.889**	**0.900**	**0.820**	**0.936**	**0.817**	0.978

Note: The best performance for each metric is marked in bold, while the second-best performance is marked in underlined.

**Figure 3:**
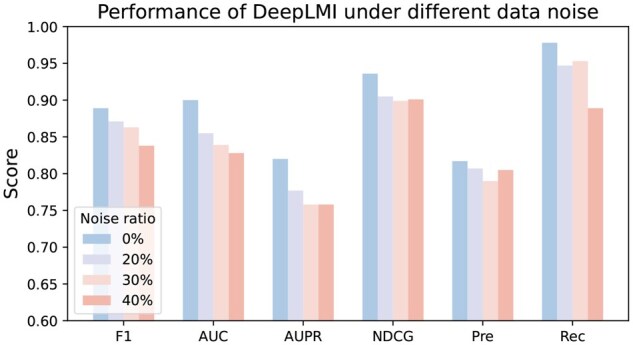
Robustness analysis of DeepLMI under increasing levels of training label noise introduced by swapping positive and negative samples.

Moreover, we performed a robustness analysis to evaluate the stability of DeepLMI under noisy training conditions, as illustrated in Fig. 3. We introduce different levels of label noise into the training data through random swapping of positive and negative labels under our independent test setting. As the noise ratio increased, a gradual decline in F1-score, AUC, and AUPR was observed, which is expected due to the reduced label reliability. Nevertheless, even under a high noise level of 40%, the model still achieved an F1 of 0.838, AUC of 0.828, and AUPR of 0.758. In addition, although recall decreased with increasing noise, precision remained relatively stable. This indicates that the model maintained high reliability in its positive predictions, despite a reduced ability to capture all positive samples under substantial label perturbation. These observations indicate that DeepLMI exhibits strong robustness to training label noise and can reliably capture underlying lncRNA–miRNA interaction patterns despite noisy annotations.

### 3.4 Ablation study

To quantify the contribution of each component in DeepLMI, we designed five variants by replacing or removing key modules. DeepLMI${\;}_{\text{onehot-mi}}$ replaces the miRNA initial node features with one-hot encoding. DeepLMI${\;}_{\text{onehot-lnc}}$ replaces the lncRNA initial node features with one-hot encoding. DeepLMI${\;}_{\text{vanilla-GCN}}$ replaces the proposed GE-GCN with a standard GCN. DeepLMI${\;}_{\text{no-in}}$ removes the interaction extraction module. DeepLMI${\;}_{\text{only-in}}$ only use lncRNA embeddings and miRNA embeddings from the interaction extraction module. Table 6 reports the ablation results under standard cross-validation setting. Overall, replacing the learned features with one-hot encodings (DeepLMI${\;}_{\text{onehot-mi}}$ and DeepLMI${\;}_{\text{onehot-lnc}}$) markedly degrades performance compared with the full DeepLMI, confirming the necessity of multisource miRNA features and multiscale lncRNA semantics. Substituting the proposed GE-GCN with a vanilla GCN (DeepLMI${\;}_{\text{vanilla-GCN}}$) also yields consistent drops, highlighting that global information exchange effectively exploits sparse interaction graphs. Further removing heterogeneous graph message passing (DeepLMI${\;}_{\text{no-in}}$) reduces AUC and AUPR to 0.918 and 0.885, showing that cross-type relational propagation is indispensable for capturing lncRNA–miRNA dependencies. Finally, the results of DeepLMI${\;}_{\text{only-in}}$ underscore the importance of extracting fine-grained information from both lncRNA and miRNA individually.

**Table 6:** Ablation results of DeepLMI under five-fold cross-validation.

Methods	F1	AUC	AUPR	NDCG	Pre	Rec
DeepLMI${}_{\text{onehot-mi}}$	0.858	0.920	0.914	0.985	0.791	0.939
DeepLMI${}_{\text{onehot-lnc}}$	0.881	0.924	0.896	0.975	0.825	0.950
DeepLMI${}_{\text{vanilla-GCN}}$	0.898	0.940	0.928	0.988	0.828	0.980
DeepLMI${}_{\text{no-in}}$	0.900	0.918	0.885	0.977	0.832	**0.982**
DeepLMI${}_{\text{only-in}}$	0.850	0.923	0.917	0.985	0.833	0.870
DeepLMI	**0.901**	**0.952**	**0.945**	**0.991**	**0.843**	0.967

Note: The best performance for each metric is marked in bold.

### 3.5 Visualization of DeepLMI embeddings

**Figure 4:**
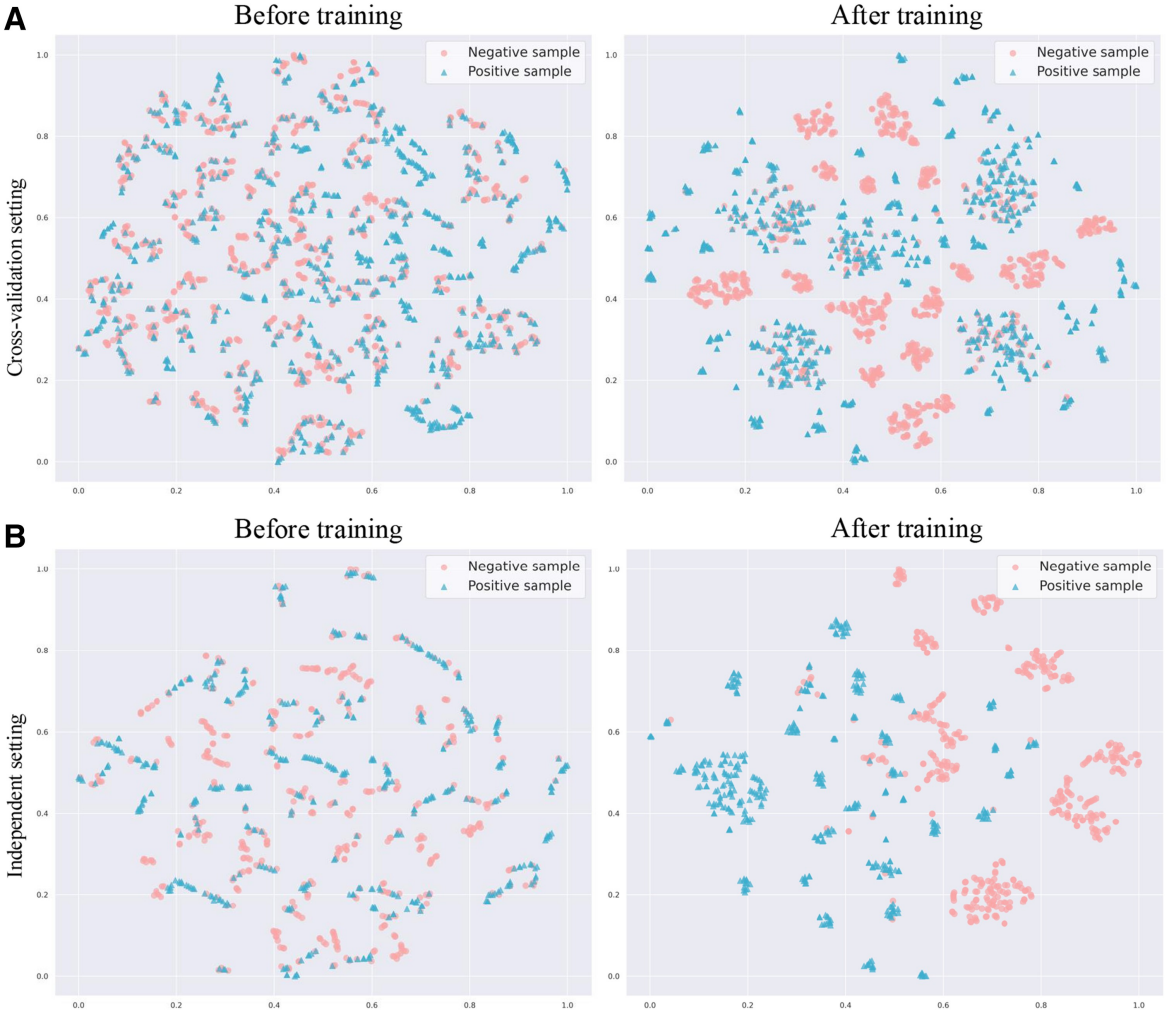
The t-SNE visualization of lncRNA–miRNA pair embeddings before and after training under (A) standard cross-validation setting and (B) independent setting.

To further illustrate that our model effectively captures the interaction patterns between lncRNA and miRNA entities, we applied t-SNE (Maaten and Hinton 2008) to perform dimensionality reduction on the learned embeddings, and then visualized the resulting 2D embeddings under two experimental settings: standard cross-validation setting and independent setting. For each setting, we plotted the concatenated embeddings of lncRNA–miRNA pairs before and after training in a 2D space. As shown in Fig. 4, before training, the embeddings are randomly distributed, with positive (interacting) and negative (noninteracting) pairs largely mixed, indicating that the model has not yet learned meaningful interaction features. After training, however, the embeddings exhibit clear structural separation: positive pairs cluster closely together, while negative pairs form distinct groups. This consistent pattern across both settings demonstrates that the model learns discriminative and generalizable representations that capture the intrinsic interaction information between lncRNAs and miRNAs.

## 4 Case study

To further verify the capability of our proposed model to predict potential lncRNA–miRNA interactions, we conducted a case study focusing on three representative lncRNAs: myocardial infarction-associated transcript (MIAT), HOX transcript antisense RNA (HOTAIR), and nuclear paraspeckle assembly transcript 1 (NEAT1). For each lncRNA, we examined the top-10 predicted miRNAs, and then validated these predictions against existing literature in PubMed.

MIAT has been implicated in cardiovascular and neurological diseases as well as multiple cancers. It functions as a ceRNA by sequestering miRNAs and thus modulating gene expression. MIAT is directly linked to myocardial infarction and other cardiovascular disorders, and it also plays roles in neurological and immune-related diseases (Li *et al.* 2018b). As shown in Table 7, five of the top-10 predicted miRNAs were supported by previous studies. For example, Huang *et al.* (2018a) reported that MIAT functions as a competing endogenous RNA by sponging miR-214 in myocardial infarction. Similarly, Li *et al.* (2018a) demonstrated that MIAT promotes tumorigenesis via regulation of miR-133a-3p. In addition, evidence exists for MIAT–miR-124-3p (Ouyang *et al.* 2022) and MIAT–miR-26b (Li *et al.* 2022) interactions, while MIAT–miR-141-3p has also been experimentally confirmed (Sha *et al.* 2018). Several other predictions, such as MIAT–miR-217 and MIAT–miR-122, currently lack validation, but may represent novel interaction candidates worthy of further exploration.

**Table 7:** The top-10 predicted results of MIAT.

Rank	miRNA	PMID
1	miR-214	29097358
2	miR-217	Unknown
3	miR-133a-3p	29772434
4	miR-101	Unknown
5	miR-124-3p	34989253
6	miR-26b	35154542
7	miR-122	Unknown
8	miR-193a	Unknown
9	miR-126	Unknown
10	miR-141-3p	29540201

HOTAIR is one of the most widely studied oncogenic lncRNAs and is known to regulate gene expression through chromatin remodeling. It is transcribed from the HOXC locus and acts as an epigenetic scaffold (recruiting PRC2) to alter chromatin state, driving metastasis and poor prognosis in multiple cancers (Zhu *et al.* 2022). In the cytoplasm, HOTAIR also acts as a ceRNA that sponges tumor-suppressive miRNAs, thereby derepressing oncogenic signaling pathways. Table 8 shows that seven of the predicted top-10 miRNAs are supported by PubMed evidence. For instance, Liu *et al.* (2019) identified HOTAIR–miR-214 as a regulatory axis in cancer progression. Additional validated interactions include HOTAIR–miR-124-3p, HOTAIR–miR-101, HOTAIR–miR-122, HOTAIR–miR-34a, HOTAIR–miR-155, and HOTAIR–miR-141-3p. These findings highlight the strong biological relevance of our predictions, while novel candidates such as HOTAIR–miR-495 and HOTAIR–miR-26b await future experimental verification.

**Table 8:** The top-10 predicted results of HOTAIR.

Rank	miRNA	PMID
1	miR-214	31933720
2	miR-124-3p	36529308
3	miR-101	28251884
4	miR-495	Unknown
5	miR-122	30195653
6	miR-34a	31325197
7	miR-26b	Unknown
8	miR-155	38339237
9	miR-141-3p	39853766
10	miR-21	39267091

More results regarding NEAT1 can be found in the Supplementary Material, available as supplementary data at *Bioinformatics* online. In total, more than half of the predicted miRNA partners for each lncRNA were supported by experimental evidence, which demonstrates the strong biological plausibility of our model’s predictions. Importantly, the remaining predictions without current validation (e.g. MIAT–miR-217, HOTAIR–miR-495) provide promising candidates for future wet-lab studies. These case studies highlight that our method not only recapitulates known interactions but also uncovers novel potential interactions, underscoring its utility in guiding experimental discovery of lncRNA–miRNA regulatory mechanisms.

## 5 Conclusion and discussion

In this study, we proposed DeepLMI, a novel deep learning framework for robust and accurate prediction of lncRNA–miRNA interactions. DeepLMI leverages tailored feature mining strategies for each RNA type—multiscale semantic embeddings for lncRNAs and multisource structural and sequence embeddings for miRNAs—enabling comprehensive representation of heterogeneous RNA characteristics. To further capture the sparse and complex interaction patterns within the lncRNA–miRNA network, we introduced a GE-GCN that integrates both local neighborhood information and long-range global dependencies. Extensive experiments under various evaluation settings, including cold-start and independent tests, demonstrate that DeepLMI consistently outperforms state-of-the-art methods. Visualization analyses further validate that DeepLMI effectively learns biologically meaningful interaction features. Overall, these findings indicate that DeepLMI provides a powerful computational tool for uncovering novel lncRNA–miRNA interactions, offering valuable support for biological discovery and the identification of potential therapeutic targets.

In future work, we will focus on enhancing model interpretability to advance the biological relevance of lncRNA–miRNA interaction predictions. Specifically, we aim to analyze internal model representations to assess the contribution of specific sequence regions and features. This interpretability-focused framework is designed not only to maintain predictive accuracy but also to uncover key regulatory patterns, thereby providing clearer mechanistic insights to support the study and experimental validation of high-confidence lncRNA–miRNA interactions.

## Supplementary Material

btag145_Supplementary_Data

## Data Availability

The codes and data underlying this article are available at https://github.com/Hhhzj-7/DeepLMI.
