# Supplementary material for: DeepLMI: deep feature mining with a globally enhanced graph convolutional network for robust lncRNA–miRNA interaction prediction
Source: Bioinformatics. 2026 Mar 26;42(4):btag145. doi: 10.1093/bioinformatics/btag145 (PMC13091651; doi:10.1093/bioinformatics/btag145)
Supplement: btag145_Supplementary_Data [file btag145_supplementary_data.pdf]

DeepLMI: Deep Feature Mining with a Globally Enhanced Graph Convolutional Network for Robust lncRNA–miRNA Interaction Prediction

Zhijian Huang [1], Kai Chen [1], Xianshu Wang [1], Junheng Wang [1], Siyuan Shen [1], Yuanpeng Zhang [1], Min Wu [2] and Lei Deng [1,*]

*To whom correspondence should be addressed.

[1]School of Computer Science and Engineering, Central South University, Changsha, 410083, China and

[2]Institute for Infocomm Research, Agency for Science, Technology and Research (A*STAR), 138632, Singapore.

Contents

1 Details of interaction prediction module 2

2 Details of datasets and experimental settings 3

3 ROC and PR curves of our model under standard setting 5

4 Evaluation metrics 6

5 Case study on NEAT1 8

1 Details of interaction prediction module

After obtaining both the biological feature embeddings and the interaction topology embeddings for lncRNA and miRNA, we designed an interaction prediction module to integrate this multi-perspective information and to score the potential interaction between lncRNA-miRNA pairs. We first fused their fine-grained embedding (E^{M}_{Lnc} and E^{M}_{Mi}) and interaction topology embedding (E^{I}_{Lnc} and E^{I}_{Mi}) by taking their mean to obtain the final comprehensive representation (E_{Lnc} and E_{Mi}):

$$E_{\text{Lnc}} = \frac{E^{M}_{\text{Lnc}} + E^{I}_{\text{Lnc}}}{2} \tag{S1}$$

$$E_{\text{Mi}} = \frac{E^{M}_{\text{Mi}} + E^{I}_{\text{Mi}}}{2} \tag{S2}$$

Then, we concatenated these two embeddings to form a combined feature vector and feed them into a Multi-Layer Perceptron (MLP):

$$s = \text{Sigmoid}(\text{MLP}([E_{\text{Lnc}} \| E_{\text{Mi}}])) \tag{S3}$$

The output is a predicted interaction score, representing the likelihood of an interaction between lncRNA and the miRNA.

2 Details of datasets and experimental settings

To ensure a robust evaluation, we assessed our model using two distinct datasets under three experimental settings. The details of two datasets can be found in Table S1. The cross-validation is constructed based on the dataset from [1], after removing redundant interaction entries to ensure higher data consistency and reliability. It comprises 284 unique lncRNAs, 517 miRNAs, and 1,057 experimentally verified lncRNA–miRNA interactions. The lncRNA sequences were sourced from LNCipedia [2] and NONCODE [3], while miRNA sequences were obtained from miRBase [4]. The interaction data were curated from LncACTdb v3.0 [5].

The external dataset was independently constructed to assess the model's generalization capability. It includes 472 lncRNAs, 295 miRNAs, and 890 interaction pairs, compiled from RNAcentral [6], NONCODE, and starBase v2.0 [7]. Unlike the cross-validation dataset, which serves primarily for training and cross-validation, the external dataset is used solely for independent testing and has no overlap with the training set.

Table S1: Overview of Cross-validation Dataset and External Dataset.

Dataset	lncRNAs	miRNAs	Interactions	lncRNA Source	miRNA Source	Interaction Source
Cross-validation Dataset	284	517	1057	LNCipedia, NONCODE	miRBase	LncACTdb v3.0
External Dataset	472	295	890	RNAcentral, NONCODE	miRBase	starBase v2.0

To comprehensively assess the predictive performanc, we designed three groups of experimental settings:

- **Setting 1: Standard cross-validation.** We conducted 5-fold cross-validation (CV) on the cross-validation dataset, ensuring that each fold maintains different lncRNA-miRNA interaction values. This setup allows efficient use of lncRNA-miRNA interaction data and reduces variance due to random partitioning, providing a more robust and unbiased assessment of model performance.
- **Group 2: Blind test on cross-validation dataset.** To simulate real-world

cold-start conditions, we constructed three distinct dataset partitions for blind testing: one based on lncRNAs, one on miRNAs, and one on both lncRNAs and miRNAs. In the blind lncRNA setting, all lncRNAs were randomly partitioned into five disjoint folds. For each fold, interactions associated with lncRNAs assigned to the validation fold were used for validation, while interactions involving the remaining lncRNAs were used for training. Under this setting, the same miRNAs may appear in both the training and validation sets, while all lncRNAs in the validation set are completely unseen during training. This setting aims to assess the model's ability to infer interactions for novel lncRNAs. The blind miRNA setting is analogous to the blind lncRNA setting, except that miRNAs in the validation set are completely unseen during training, while lncRNAs may appear in both training and validation sets. In the blind both setting, lncRNAs and miRNAs were independently divided into five folds. Consequently, neither lncRNAs nor miRNAs in the validation set appear in the training data. This setting represents a fully cold-start scenario and provides a rigorous evaluation of the model's robustness and its ability to generalize to completely unseen lncRNA–miRNA interaction.

- **Group 3: Independent Test.** To evaluate model robustness across data sources, the model was trained on the cross-validation dataset and subsequently validated and tested on the external dataset. It is important to note that these two datasets were sourced from disjoint sources to ensure a rigorous evaluation. This setting reflects a realistic deployment scenario where the model must generalize to completely new data source.

3 ROC and PR curves of our model under standard setting

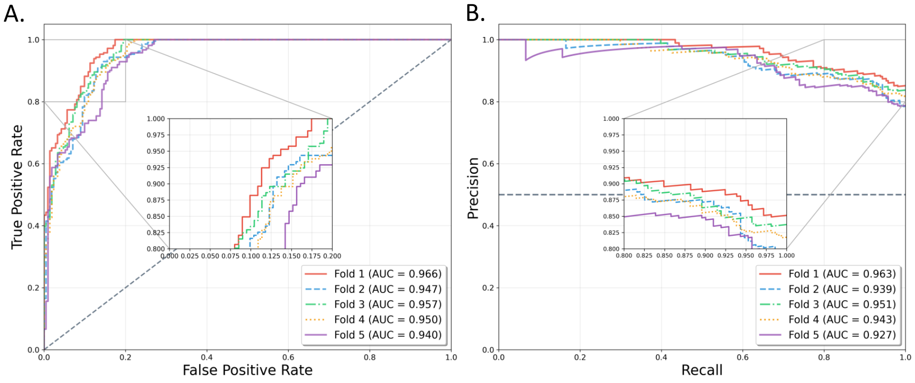

Figure S1: ROC and PR curves of our model under standard cross-validation setting. (A) is ROC curve and (B) is PR curve.

Fig. S1 shows the ROC and PR curves of our model under standard setting. The curves of all five folds exhibit highly consistent trends, indicating that the model performs robustly across different data partitions. This consistency suggests that DeepLMI does not rely on specific subsets of the data to achieve good performance, but instead captures generalizable patterns. Such stability across folds further demonstrates the reliability of our training procedure and the overall robustness of our proposed method.

4 Evaluation metrics

To comprehensively evaluate the predictive performance of the proposed model, we employed six widely used metrics, including AUC, AUPR, NDCG, Precision, Recall, and F1-score, which assess model performance from complementary perspectives. Precision measures the proportion of correctly predicted positive samples among all predicted positives and is defined as

$$\text{Precision} = \frac{TP}{TP + FP}, \tag{1}$$

where TP and FP denote the numbers of true positives and false positives, respectively.

Recall quantifies the proportion of true positive samples that are correctly identified and is defined as

$$\text{Recall} = \frac{TP}{TP + FN}, \tag{2}$$

where FN denotes the number of false negatives.

The F1-score is the harmonic mean of Precision and Recall, providing a balanced evaluation of classification performance, and is defined as

$$\text{F1} = \frac{2 \times \text{Precision} \times \text{Recall}}{\text{Precision} + \text{Recall}}. \tag{3}$$

In addition to threshold-dependent metrics, we adopted several threshold-independent and ranking-based metrics to provide a more comprehensive evaluation. The area under the receiver operating characteristic curve (AUC) measures the overall discriminative ability of the model by estimating the probability that a randomly selected positive sample is ranked higher than a randomly selected negative sample. It is defined as

$$\text{AUC} = \int_0^1 \text{TPR}(x)\, d\,\text{FPR}(x), \tag{4}$$

where TPR and FPR denote the true positive rate and false positive rate, respectively.

The area under the precision–recall curve (AUPR) summarizes the trade-off between Precision and Recall over all possible decision thresholds and is defined as

$$\text{AUPR} = \int_0^1 \text{Precision}(r)\, d\,\text{Recall}(r). \tag{5}$$

AUPR focuses on the model's ability to correctly identify positive interactions.

Finally, the normalized discounted cumulative gain (NDCG) is employed to evaluate the quality of ranking results, emphasizing the importance of correctly ranking true interactions at top positions. For a ranked list of length K, NDCG is defined as

$$\mathrm{NDCG@}K = \frac{1}{\mathrm{IDCG@}K} \sum_{i=1}^{K} \frac{2^{rel_i} - 1}{\log_2(i+1)},$$

where rel_i denotes the relevance score of the item at position i, and $\mathrm{IDCG@}K$ represents the ideal discounted cumulative gain.

5 Case study on NEAT1

NEAT1 is another lncRNA that plays crucial roles in cancer biology and other diseases. NEAT1 is consistently upregulated in many tumors (breast, prostate, lung, colorectal, etc.) and generally acts as an oncogene by promoting proliferation and metastasis [8]. It sponges multiple miRNAs that would otherwise suppress oncogenic mRNAs. As shown in Table S2, six of the predicted top-10 miRNAs are supported by published studies. For example, NEAT1–let-7a-5p has been validated in tumor regulation [9], while NEAT1–miR-17-5p, NEAT1–let-7c-5p, NEAT1–miR-143, NEAT1–let-7b-5p, and NEAT1–miR-153 are also well documented. Other predicted pairs, such as NEAT1–miR-183 and NEAT1–miR-30b, are not yet reported, but may represent novel interactions with potential biological significance.

Table S2: The top-10 predicted results of NEAT1.

Rank	miRNA	PMID
1	hsa-let-7a-5p	39484776
2	miR-183	Unknown
3	miR-30b	Unknown
4	miR-106a	Unknown
5	miR-17-5p	32099901
6	hsa-let-7c-5p	38429903
7	hsa-let-7e-5p	Unknown
8	miR-143	33744906
9	hsa-let-7b-5p	39919453
10	miR-153	39422618

References

[1] Zixiao Wang, Shiyang Liang, Siwei Liu, Zhaohan Meng, Jingjie Wang, and Shangsong Liang. Sequence pre-training-based graph neural network for predicting lncrna-mirna associations. *Briefings in Bioinformatics*, 24(5):bbad317, 2023.

[2] Pieter-Jan Volders, Jasper Anckaert, Kenneth Verheggen, Justine Nuytens, Lennart Martens, Pieter Mestdagh, and Jo Vandesompele. Lncipedia 5: towards a reference set of human long non-coding rnas. *Nucleic acids research*, 47(D1):D135–D139, 2019.

[3] Lianhe Zhao, Jiajia Wang, Yanyan Li, Tingrui Song, Yang Wu, Shuangsang Fang, Dechao Bu, Hui Li, Liang Sun, Dong Pei, et al. Noncodev6: an updated database dedicated to long non-coding rna annotation in both animals and plants. *Nucleic acids research*, 49(D1):D165–D171, 2021.

[4] Ana Kozomara, Maria Birgaoanu, and Sam Griffiths-Jones. mirbase: from microrna sequences to function. *Nucleic acids research*, 47(D1):D155–D162, 2019.

[5] Peng Wang, Qiuyan Guo, Yue Qi, Yangyang Hao, Yue Gao, Hui Zhi, Yuanfu Zhang, Yue Sun, Yakun Zhang, Mengyu Xin, et al. Lncactdb 3.0: an updated database of experimentally supported cerna interactions and personalized networks contributing to precision medicine. *Nucleic acids research*, 50(D1):D183–D189, 2022.

[6] RNAcentral Consortium. Rnacentral 2021: secondary structure integration, improved sequence search and new member databases. *Nucleic acids research*, 49(D1):D212–D220, 2021.

[7] Jun-Hao Li, Shun Liu, Hui Zhou, Liang-Hu Qu, and Jian-Hua Yang. starbase v2. 0: decoding mirna-cerna, mirna-ncrna and protein–rna interaction networks from large-scale clip-seq data. *Nucleic acids research*, 42(D1):D92–D97, 2014.

[8] Jingtao Gu, Bo Zhang, Rui An, Weikun Qian, Liang Han, Wanxing Duan, Zheng Wang, and Qingyong Ma. Molecular interactions of the long noncoding rna neat1 in cancer. *Cancers*, 14(16):4009, 2022.

[9] Ali Samareh, Mohammad Hadi Nematollahi, Hossein Pourghadamyari, Hossein Ali Ebrahimi Meimand, Mohammad Shabani, and Gholamreza Asadikaram. Diagnostic potential of neat1, hsa-let-7a-5p, and mir-506-3p in early-stage parkinson's disease. *Current Medicinal Chemistry*, 32(36):8214–8228, 2025.
